# Children's Country Holidays Fund

**Published:** 1917-05-05

**Authors:** Francis Morris

**Affiliations:** Chairman of the Fund. 18 Buckingham Street, Strand, W.C. 2.


### Children's Country Holidays Fund.

*To the Editor of* THE HOSPITAL.

SIR,—In order to minimise expenditure the Council of the Children's Country Holidays Fund is holding no annual meeting of subscribers this year. I should be glad therefore of an opportunity of announcing through your columns that, although increasing difficulties of work will enforce a further reduction in the number of children to be sent away, careful effort is being made to secure a country holiday for a proportion of the most ailing children in the elementary schools.

One can hardly overrate the value of country air and country rest to the health of these children. In normal times the Fund stands for the doctrine that a real country holiday, in the sense of a complete escape from the drab, crowded, everyday existence, is to be desired for every poor London child; in wartime one can only concentrate on those whose health most imperatively demands the change.

The total cost of the holiday has increased very considerably owing to the higher payments necessary in the country, and subscriptions are needed for this year's work.

Cheques made payable to the Fund should be sent to the Secretary, 18 Buckingham Street, Strand, W.C.—Yours faithfully,

FRANCIS MORRIS, Chairman of the Fund.

18 Buckingham Street, Strand, W.C. 2., April 26, 1917.

